# Comparative Analysis of Adhesive Strength and Flexibility in Surgical Sealants for Cardiovascular Surgery

**DOI:** 10.3400/avd.oa.25-00100

**Published:** 2026-01-07

**Authors:** Akiyoshi Yamamoto, Shinichiro Shimura, Kenji Kuwaki, Hidekazu Furuya, Sohsyu Kotani, Kimiaki Okada, Keisuke Ozawa, Goro Kishinami, Shigeyuki Ozaki, Yasunori Cho

**Affiliations:** 1 Department of Cardiovascular Surgery, Tokai University School of Medicine, Isehara, Kanagawa, Japan; 2 Department of Cardiovascular Surgery, Toho University Ohashi Medical Center, Tokyo, Japan; 3 Department of Cardiovascular Surgery, Tokai University Hachioji Hospital, Hachioji, Tokyo, Japan

**Keywords:** Hydrofit, BioGlue, Beriplast, adhesive strength test, flexibility test

## Abstract

**Objectives:**

The objective of this study was to compare the adhesive strength and flexibility of 3 surgical sealants—synthetic (Hydrofit; Sanyo Chemical Industries, Kyoto, Japan), albumin-based (BioGlue; Artivion, Kennesaw, GA, USA), and fibrin-based (Beriplast; CSL Behring, Tokyo, Japan)—which are commonly used in cardiovascular surgery but unexplored under identical experimental conditions.

**Methods:**

Adhesive strength was evaluated using a tensile adhesion test on collagen, polyester, and polytetrafluoroethylene substrates. Flexibility was assessed by measuring the maximum stress and elongation at failure in Hydrofit and BioGlue film samples. Beriplast was excluded as it failed to form films.

**Results:**

Hydrofit and BioGlue showed similar collagen–collagen adhesion strengths (p = 0.11), while Beriplast was significantly weaker (p <0.01). Hydrofit outperformed both BioGlue and Beriplast (p <0.01) in collagen–polyester and collagen–expanded polytetrafluoroethylene (ePTFE) adhesions. Hydrofit also demonstrated a significantly higher elongation rate, strength, and maximum stress before rupture than BioGlue.

**Conclusions:**

These surgical sealants possess distinct adhesive and mechanical characteristics. Hydrofit showed stable adhesion across various substrates, with notable flexibility. BioGlue displayed adequate adhesion on collagen surfaces but had restricted flexibility. Beriplast demonstrated reduced adhesion. Although only adhesive strength and flexibility were evaluated, such properties may offer valuable insights into sealant traits contextually. These potentially aid in the selection of appropriate sealants for cardiovascular procedures that require both durable adhesion and tissue compliance. Further *in vivo* validation is warranted.

## Introduction

Surgical hemostatic agents are occasionally utilized not only for their hemostatic properties but also as tissue adhesives. For example, in aortic dissection repair, these agents may be applied to seal the false lumen by bonding the dissected aortic layers. In such cases, the mechanical properties of the agent—particularly its adhesive strength and elasticity—are believed to play a crucial role in ensuring the durability and integrity of the repair over time.^1,2)^

Despite their widespread clinical use, the adhesive and elastic properties of these agents have not been quantitatively or systematically compared. This lack of data represents a significant gap in the literature, particularly given the increasing reliance on such materials in complex cardiovascular procedures. Understanding the mechanical behavior of hemostatic agents under conditions that mimic surgical use is essential for selecting the most appropriate material and optimizing surgical outcomes.

The present study aims to address this gap by conducting a comparative evaluation of the mechanical properties—specifically adhesive strength and elasticity—of commonly used surgical hemostatic agents. Through this investigation, we seek to provide foundational data that may inform material selection and improve long-term surgical durability in aortic and other vascular repairs.

In cardiac surgery, achieving hemostasis is vital for reducing surgical mortality and morbidity; however, it can be challenging to accomplish. This is due to cardiopulmonary bypass with systemic heparinization and reduced coagulation from hypothermia.^3,4)^ Therefore, surgical sealants are used to control bleeding in surgical hemostasis.^5–7)^ Various surgical sealants (biological and nonbiological) are now commercially available. However, adhesive strength is a significant function of surgical sealants, which are also used for adhesive purposes,^8,9)^ such as sealing the false lumen in aortic dissection.^2,10,11)^ Additionally, the differences in elasticity among surgical sealants should be assessed for their compatibility with physical tissues. Surgical sealants vary in tissue adhesion and elasticity. In this study, we focused on these properties by comparing the characteristics of 3 surgical sealants representing different categories—synthetic (Hydrofit; Sanyo Chemical Industries, Kyoto, Japan), albumin-based (BioGlue; Artivion, Kennesaw, GA, USA), and fibrin-based (Beriplast; CSL Behring, Tokyo, Japan)—commonly applied in cardiovascular surgery. To date, no studies have reported such a comparison under similar conditions.

We assessed the adhesive strength and flexibility of Hydrofit, BioGlue, and Beriplast—widely used surgical sealants in clinical practice. Adhesion tests were conducted using collagen, polyester, and polytetrafluoroethylene (PTFE) substrates in a moist, simulated surgical environment, ensuring compliance with the instructions for use (IFU) of each agent. Flexibility was assessed by measuring the elongation and stress properties of the Hydrofit and BioGlue film samples.

## Materials and Methods

Three surgical sealants were used in this study: Hydrofit, BioGlue, and Beriplast. Hydrofit is a urethane resin-forming prepolymer that cures upon reaction with water, thereby polymerizing the sealant. The diisocyanate-coated polyol reacts with water to form isocyanate-amino groups that release carbon dioxide (Eq. (1)). Polymerization through a strong addition reaction between amino groups and unreacted isocyanate groups yields an elastic polyurethane polymer (Eq. (2))

(1) $$\begin{array}{l} \text{OCN–R–NCO}+{\text{H}}_{2}\text{O}\to \left[\text{HO–CO–NH–R–NCO}\right]\\ \to {\text{H}}_{2}\text{N–R–NCO}+{\text{CO}}_{2} \end{array}$$

(2) $$\begin{array}{l} \text{nOCN–R–NCO}+{\text{H}}_{2}\text{N–R–NCO}+\left(\text{n}+\text{1}\right){\text{ H}}_{2}\text{O}\to \\ {\text{H}}_{2}\text{N–R–}{\left(\text{NHCO–NH–R}\right)}_{\text{n}}{\text{–NH}}_{2}+\left(\text{n}+1\right)\;{\text{CO}}_{2} \end{array}$$

BioGlue is a strong surgical adhesive in which 2 solutions—45% bovine serum albumin (BSA) and glutaraldehyde—are filled in a syringe. Pressing the plunger mixes the 2 solutions, causing glutaraldehyde-mediated crosslinking of *in vivo* tissue proteins and BSA, which reaches its maximum strength in approximately 2 min. Beriplast consists of human fibrinogen, coagulation factor XIII, thrombin, calcium ions, and aprotinin, which protects the fibrin clot from fibrinolytic enzymes.

Collagen, polyester, and PTFE were used as adherends in the tests. A collagen sheath (#320; Nippi, Tokyo, Japan) was used as a collagen substitute after water treatment and subsequent drying. A surgical vascular graft (Gelweave graft; Terumo, Tokyo, Japan) was used as polyester, and PTFE felt (Matsuda Medical Industries, Tokyo, Japan) was used as PTFE.

### Adhesive strength test

The adhesive strength was measured using a tensile adhesion test on an Autograph (AGX-10kNVD RY; Shimadzu, Kyoto, Japan) with collagen, polyester, and PTFE substrates. Each adherend material was cut into 1.0 × 4.0 cm^2^ pieces, moistened with ion-exchanged water, wiped with Kimwipes (Nippon Paper Crecia, Tokyo, Japan) paper, and laminated at a 1 cm edge with each hemostatic agent. The applied amounts were approximately 200 µg of Hydrofit, 14.8 µL of albumin and 3.7 µL of glutaraldehyde for BioGlue, and 10 µL each of Beriplast solutions A and B. The application procedures followed the IFU. After application, a 100 g weight was placed and pressed for the specified duration—Hydrofit (5 min), BioGlue (2 min), and Beriplast (5 min)—based on the IFU. After securing both ends of the specimen to the testing machine, one end was pulled at a speed of 300 mm/min. The stress at detachment was measured to determine the bond strength. A total of 21 specimens were tested, 7 for each hemostatic agent. This test was performed in accordance with the international standard ISO 4587:1995 (Adhesives—Determination of tensile lap-shear strength of rigid-to-rigid bonded assemblies). The use of this standardized procedure ensured the reproducibility and reliability of the adhesive strength measurements conducted under moist, simulated surgical conditions.

### Flexibility test

Flexibility was confirmed by forming films of Hydrofit and BioGlue and measuring their extensibility using an Autograph. Beriplast was excluded because it failed to form a film. Each hemostatic agent was cast into a 300-μm-thick film. The specimens were punched using a No. 3 dumbbell (JIS-compliant standard). Flexibility was assessed by measuring the maximum stress at fracture and the elongation, calculated as the stroke length, while pulling one end of the specimen at 500 mm/min with the other end fixed to the testing machine. A total of 14 specimens were tested, 7 for each hemostatic agent. The flexibility test was conducted in accordance with ISO 37:2011 (Rubber, vulcanized or thermoplastic—Determination of tensile stress–strain properties). By following this standardized testing protocol, the reproducibility, reliability, and comparability of the flexibility measurements were ensured.

### Statistical analysis

Adhesive strength and flexibility are expressed as the mean ± SD (standard deviation) and were assessed using an unpaired t-test. An F-test was used to assess the equality of variances. After confirming equal variances (F-test value >0.05), an equal variance t-test was performed. The common SD for both groups was calculated, and a sample size of 7 was considered adequate. The sample size was determined based on a power analysis for detecting the difference between 2 group means using a 2-sample t-test. The following parameters were applied: a confidence level of 0.95 (α error = 0.05), a statistical power of 0.8 (1 − β = 0.8), and an equal sample size ratio of 1:1. The analysis indicated that a minimum of 6 samples per group would be sufficient to achieve adequate statistical power under these conditions. Accordingly, a slightly larger sample size of 7 specimens per group was selected to ensure robustness of the results. Statistical significance was set at p <0.05. All statistical analyses were performed using R version 4.3.3 (The R Foundation for Statistical Computing, Vienna, Austria).

## Results

### Adhesive strength test

**Figure 1:**
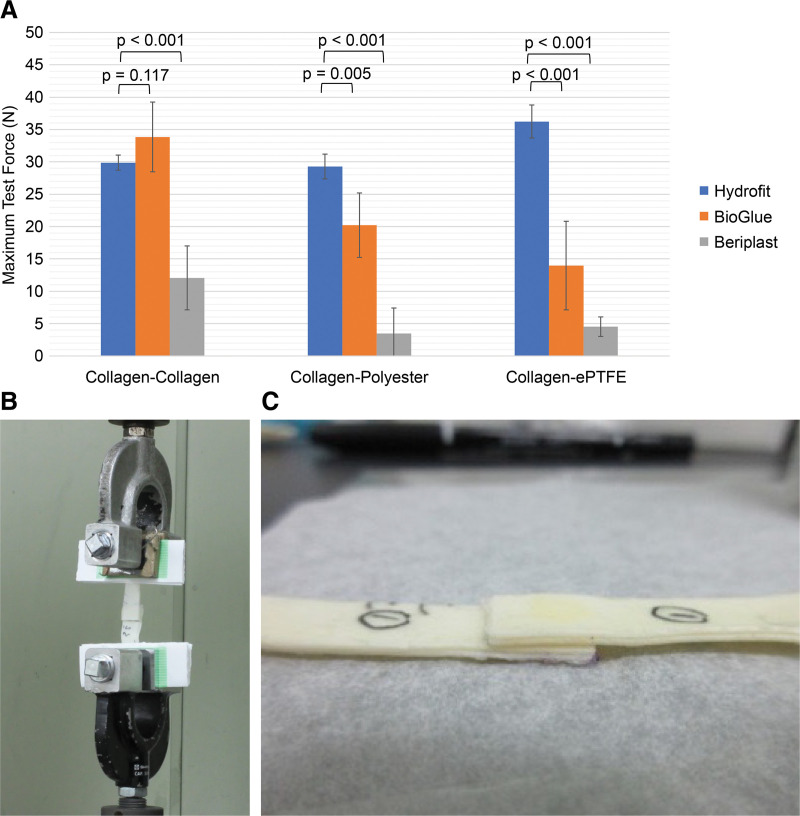
Fig. 1 Comparison of adhesive strength, traction, and performance for the chosen substrates. (**A**) Adhesive strength of Hydrofit (Sanyo Chemical Industries, Kyoto, Japan), BioGlue (Artivion, Kennesaw, GA, USA), and Beriplast (CSL Behring, Tokyo, Japan) on various substrates. Hydrofit exhibited superior adhesive strength across all tested substrates compared to other agents. (**B**) Traction tests demonstrating the stress required for the detachment of adhesives on different substrates. Hydrofit exhibited stable adhesion under applied forces. (**C**) Adhesive performance comparison of Hydrofit against BioGlue and Beriplast in collagen, polyester, and polytetrafluoroethylene substrates, highlighting significant differences. ePTFE: expanded polytetrafluoroethylene

The properties of each hemostatic agent were compared for each adherend (**Table 1**). For collagen–collagen, Hydrofit (29.89 ± 3.09 N) and BioGlue (33.86 ± 5.39 N) demonstrated no significant difference (p = 0.117), whereas Beriplast (12.07 ± 4.94 N) had significantly lower adhesion strength than Hydrofit (p <0.001). For collagen–polyester, Hydrofit exhibited high adhesion strength (29.29 ± 5.07 N), whereas BioGlue’s was slightly lower (20.22 ± 4.98 N) and Beriplast’s was significantly lower (3.49 ± 3.92 N). Both differences were statistically significant (p <0.05). For collagen–expanded PTFE (**Figs. 1A**–**1C**), the adhesion strength of Hydrofit was 36.24 ± 6.75 N, whereas those of BioGlue (13.97 ± 6.84 N) and Beriplast (4.53 ± 1.51 N) were significantly lower than that of Hydrofit (p <0.001).

**Table 1:** Results of the adhesive strength test

Adherend		Sealant	Maximum test force (N)		t-Test (vs. Hydrofit)
Sample 1	Sample 2		Mean	SD	
Collagen	Collagen	Hydrofit (n = 7)	29.89	3.09	—
		BioGlue (n = 7)	33.86	5.39	0.117
		Beriplast (n = 7)	12.07	4.94	<0.001
Collagen	Polyester	Hydrofit (n = 7)	29.29	5.07	—
		BioGlue (n = 7)	20.22	4.98	0.005
		Beriplast (n = 7)	3.49	3.92	<0.001
Collagen	PTFE	Hydrofit (n = 7)	36.24	6.75	—
		BioGlue (n = 7)	13.97	6.84	<0.001
		Beriplast (n = 7)	4.53	1.51	<0.001

*p <0.05 was considered statistically significant.

Adhesive strength of Hydrofit (Sanyo Chemical Industries, Kyoto, Japan), BioGlue (Artivion, Kennesaw, GA, USA), and Beriplast (CSL Behring, Tokyo, Japan) on various substrates (collagen, polyester, and polytetrafluoroethylene). Hydrofit consistently exhibited superior adhesive strength compared to other surgical sealants, with significant differences observed in most comparisons.

SD: standard deviation; vs: versus; PTFE: polytetrafluoroethylene

### Flexibility test

**Figure 2:**
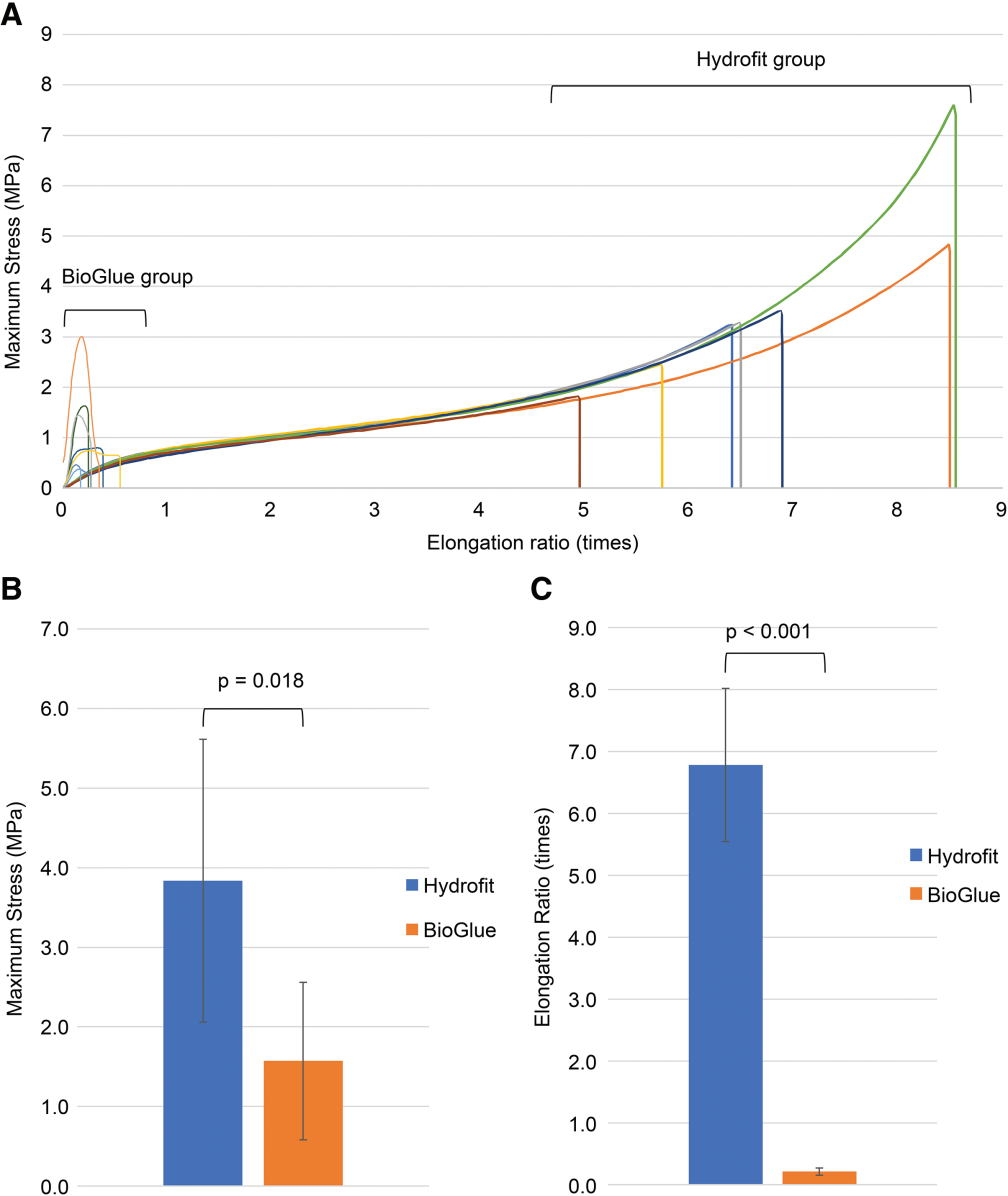
Fig. 2 Comparison of the flexibility test among the chosen substrates. (**A**) Relationship between elongation and stress during flexibility testing. Hydrofit (Sanyo Chemical Industries, Kyoto, Japan) exhibited greater elongation and stress resistance than BioGlue (Artivion, Kennesaw, GA, USA). (**B**) Maximum stress comparison: Hydrofit films exhibited significantly higher maximum stress values than BioGlue. (**C**) Elongation rate comparison: Hydrofit exhibited a significantly higher elongation rate than did BioGlue, demonstrating superior flexibility.

Beriplast was excluded from the study due to its inability to form a film. Flexibility testing (**Table 2**) demonstrated that Hydrofit exhibited significantly greater mean stroke length (135.6 mm) compared to BioGlue (4.2 mm). Hydrofit film exhibited significantly higher elongation and strength than BioGlue film. Hydrofit film exhibited significantly greater elongation (up to approximately 8.5 times) and achieved higher stress levels (up to nearly 9 MPa), indicating superior flexibility and tensile strength. In contrast, BioGlue film fractured at an early stage of elongation (approximately 0.2–0.3 times) with lower maximum stress values (**Fig. 2A**). The maximum stress for Hydrofit (3.84 ± 1.78 MPa) was significantly stronger than that of BioGlue (1.57 ± 0.99 MPa) (**Fig. 2B**). The elongation rate of Hydrofit was 7.78 times, much higher than that of BioGlue (1.21 times) (**Fig. 2C**).

**Table 2:** Results of the flexibility tests

Sealants	Mean stroke (mm)	Maximum point stress (MPa)		t-Test (vs. Hydrofit)	Elongation ratio (times)		t-Test (vs. Hydrofit)
		Mean	SD		Mean	SD	
Hydrofit (n = 7)	135.6	3.84	1.78	—	6.78	1.24	—
BioGlue (n = 7)	4.2	1.57	0.99	0.018	0.21	0.06	<0.001

*p <0.05 was considered statistically significant.

Flexibility test results of Hydrofit (Sanyo Chemical Industries, Kyoto, Japan) and BioGlue (Artivion, Kennesaw, GA, USA) films. Hydrofit exhibited significantly higher elongation and maximum stress values than those of BioGlue, highlighting its superior flexibility for surgical applications.

SD: standard deviation; vs.: versus

## Discussion

Various surgical sealants with differing tissue adhesion and elasticity have been developed and are available for clinical use. This study evaluated the adhesive strength and flexibility of Hydrofit, BioGlue, and Beriplast—3 widely used surgical sealants in clinical practice. To date, no direct comparative studies have been reported under identical experimental conditions. Numerous biologically derived surgical sealants are intended for application in dry surgical fields. However, in cardiovascular surgery, achieving a completely dry operative site is seldom feasible. In this study, each adhesive material was moistened with ion-exchanged water and wiped with Kimwipes to simulate a moist surgical environment while ensuring compliance with the IFU for each agent.

The adhesive strength was assessed using the adhesive strength test. Each hemostatic agent exhibited distinct adhesive properties under experimental conditions. Beriplast exhibited weak adhesive strength. Additionally, Beriplast alone failed to form a stable film. Its adhesion mechanism is based on fibrinogen adsorption onto the tissue, followed by a stable fibrin clot formation through the action of thrombin, Ca^2+^, and factor XIII.^12)^ The adhesive strength of fibrin glue is significantly lower than that of gelatin–resorcinol–formaldehyde glue; therefore, using Teflon felt is recommended to reinforce the aorta when using fibrin glue.^10)^ BioGlue, which adheres through a glutaraldehyde–albumin crosslinking reaction, exhibited strong adhesion on collagen–collagen substrates. This reaction induces protein denaturation, ensuring firm adhesion; however, it is also associated with a certain degree of tissue degeneration.^10)^ Zientara et al. measured the peel force of BioGlue using the T-peel test, which assesses adhesive peel strength by measuring the force required to separate tissues glued by T-peel. Applying pressure to an aortic specimen increased the peel force.^11)^ However, BioGlue exhibited limited adhesion to non-biological tissues, and in our experiments, certain specimens detached before being affixed to the device, similar to Beriplast. Hydrofit, a synthetic elastomeric adhesive, demonstrated strong adhesion to both biological and non-biological tissues through interlocking mechanisms and direct chemical bonding, resulting in stable and durable bonding.^13)^ Hydrofit has been tested in adult canine femoral and carotid arteries and has demonstrated effective tissue adhesion, hemostasis at the anastomotic sites, and long-term patency.^13–15)^

The flexibility of each adhesive—relevant for use in vascular and cardiac tissues—was assessed. Differences in the mechanical properties of the 3 agents were observed. BioGlue became rigid and brittle after curing. This loss of flexibility may limit its use in applications that require tissue compliance and elasticity. In contrast, Hydrofit maintained a relatively flexible structure after curing, forming an elastic film. Owing to its inability to form a film, Beriplast was excluded from this experiment. After film formation, BioGlue exhibited low elongation and low stress resistance, making it more prone to fracture under mechanical load. In contrast, Hydrofit exhibited both higher elongation and resistance to traction forces (**Supplementary Video 1**). These differences in flexibility and mechanical behavior align with previous findings.^16)^ Hydrofit maintains its elastomeric properties and forms a stable, flexible seal^17)^ that helps minimize stress concentration at the anastomotic site, specifically under high-pressure, pulsatile arterial flow.^7)^ For false lumen closure of the aortic root in acute aortic dissection surgery, Hydrofit may offer advantages over BioGlue by preserving the physiologic expansion and contraction of the vessel wall during cardiac pulsation, including hemodynamic responsiveness, due to its potentially greater tissue flexibility. However, this remains a hypothesis and warrants further investigation. Gross examination and histopathology demonstrated no inflammation or tissue infiltration around the Hydrofit removed over 4 years after implantation.^18)^ In contrast, long-term complications related to BioGlue have been reported, specifically the formation of late postoperative aortic root pseudoaneurysms attributed to chronic inflammation and tissue degeneration.^19–21)^ Although BioGlue has been officially approved for clinical use in aortic dissection in Japan, concerns remain regarding the formation of pseudoaneurysm, as reported in previous studies.^19–21)^ Therefore, we consider that careful use and long-term evaluation of its clinical outcomes are warranted. These findings underscore the need for further clinical studies to better understand the long-term biocompatibility and safety profiles of surgical sealants used in aortic procedures. Although the present findings are limited to the evaluation of adhesive strength and flexibility, these mechanical properties are important factors to be considered when selecting surgical sealants, depending on the procedural context. Previous studies have reported that Hydrofit and BioGlue, owing to their strong adhesive properties, can effectively promote hemostasis and tissue bonding at the anastomotic interface between the aorta and vascular grafts in aortic surgery, as well as at the myocardial suture line during repair of cardiac rupture. Hydrofit has demonstrated reliable hemostatic efficacy and durable anastomotic sealing in both experimental and clinical cardiovascular settings, even under full heparinization, and has also been successfully utilized for the management of cardiac rupture.^13,16,22)^ Similarly, BioGlue has been employed as an adjunctive hemostatic agent in aortic surgery and for reinforcement of myocardial repair following cardiac rupture.^1,10,23,24)^ However, in coronary anastomoses during coronary artery bypass grafting (CABG), excessive adhesive strength may result in stenosis or occlusion at the graft anastomotic site. Both animal experiments and clinical observations have indicated that highly adhesive glutaraldehyde-based sealants can induce luminal narrowing or embolic complications when applied near coronary anastomoses.^25,26)^ In such cases, a fibrin-based sealant, such as the Beriplast spray method, may represent a preferable topical hemostatic adjunct, as it minimizes the risk of luminal compromise and has been reported to reduce postoperative bleeding following CABG.^27,28)^

Further studies are required to determine the optimal application conditions for each surgical sealant and to assess their long-term biocompatibility and durability *in vivo*.

## Conclusion

This study evaluated the adhesive strength and flexibility of 3 commonly used surgical sealants—Hydrofit, BioGlue, and Beriplast—under identical experimental conditions. The results indicate that the tested sealants demonstrated varying adhesive and mechanical properties. Hydrofit showed consistent adhesion across all tested substrates and retained its flexibility after fixation. BioGlue provided strong adhesion on collagen surfaces but had relatively limited flexibility, whereas Beriplast demonstrated lower adhesion and lacked consistency in forming a stable film. Although this study focused exclusively on adhesive strength and flexibility, these characteristics may provide valuable insights into the properties of surgical sealants in relation to procedural requirements. These mechanical profiles may aid in the selection of appropriate sealants for cardiovascular procedures that require both durable adhesion and tissue compliance. Further *in vivo* studies are warranted to validate these findings.

## Additional Remarks

This work was presented at the 52nd Annual Meeting of the Japanese Society for Vascular Surgery (Oita, Japan, 2024).

## Supplementary Information

Supplementary Video 1 Mechanical properties of BioGlue and Hydrofit after film formation. BioGlue is prone to fracture under mechanical load, whereas Hydrofit demonstrates higher elongation and traction resistance.
